# Optometry students’ experiences of their clinical training: A qualitative study in a low- resource setting

**DOI:** 10.21203/rs.3.rs-3993765/v1

**Published:** 2024-03-01

**Authors:** Boaz Mucunguzi, Walker Guti, Moreen Tumwine, Aloysius G. Mubuuke, Ian Munabi, Arild Raaheim, Sarah Kiguli

**Affiliations:** Makerere University; Makerere University; Kampala International University; Makerere University; Makerere University; University of Bergen; Makerere University

**Keywords:** Optometry, clinical training, clinical learning environment, low resource setting

## Abstract

**Background:**

There is a gradual increase in the number of optometry education programs in low resource settings yet there is limited knowledge on optometry students’ experiences of their clinical training. Therefore, the purpose of this study was to explore the optometry students’ experiences of their clinical learning environment at a national referral and teaching hospital within a low resource setting.

**Methods:**

The study adopted a qualitative design using face to face in-depth interviews to explore experiences of the participants. All 16 optometry students in fourth-year at university were purposefully recruited into the study. Data was collected at the end of the students’ clinical training at the eye clinic of a national referral and teaching hospital. Interviews were audio recorded and transcribed for analysis using an inductive thematic approach.

**Results:**

Two themes, learning at the eye clinic and organization of the eye clinic, were identified to represent participants’ experiences. Each theme had three sub themes.

**Conclusion:**

The students’ experiences in a clinical learning environment take a transformative nature from initial hesitancy and feelings of inferiority, anxiety, uncertainty and nervousness to increased confidence and active engagement. Future studies should compare optometry students’ experiences in lower-level health units to those in national referrals hospitals.

## BACKGROUND

Visual impairment is an increasing global challenge with cataracts and uncorrected refractive errors as the two main causes [1]. Within low resource settings (LRSs), this challenge is intensified by the shortage of human resource in eye health [2]. Between 2017 and 2020, Naidoo and colleagues mapped the global optometry workforce and revealed big differences in practitioner to population ratios in developing countries. In central sub-Saharan Africa and eastern sub-Saharan Africa, the ratios of optometrists to the population were 1: 1198141 and 1: 103441 respectively [3] compared to the recommended 1: 50000 by the World Health Organization [4]. To address this challenge, global initiatives encourage training and development of human resources for eye health [5].

On the continent of Africa, optometry education and the profession have evolved increasingly in recent years [6] with the establishment of 16 new optometry education programs over the last decade [7]. In Uganda, the program is currently offered in one institution, Makerere University College of Health Sciences (MakCHS), leading to an award of a Bachelor of Optometry degree attained after four years of training. The Bachelor of Optometry degree curriculum encompasses theoretical, practical and clinical modules [8]. Clinical learning (CL) is achieved mostly in hospital eye clinics by students interacting with patients under supervision [9]. A conducive clinical learning environment (CLE) is vital for optometry students to attain the necessary clinical competency that will enable them to practice as independent professionals [10].

Within LRSs, optometrists are significant health professionals in the eye health sector not only because of their role in treating uncorrected refractive errors, but also, they are of great importance in settings where there are no or few ophthalmologists [6, 8]. Optometry practice extends beyond treating refractive errors to prescribing drugs to treat a wide range of anterior segment diseases, and also screening for vision threats such as cataracts, glaucoma and retinal degeneration [11]. With the gradual increase in the number of optometry programs and graduating optometrists, as well as their role in eye health service delivery, it is important to understand their clinical training (CT). However, there is limited knowledge on optometry students’ experiences of the CLE within LRSs.

Learning in clinical settings involves interactions with staff, supervisors, patients, and other allied health and medical students [9]. For maximum educational value, data concerning the students’ experiences in the CLE is needed to effectively respond to changing needs in both clinical education and patient care [10]. Also, knowledge of students’ experiences of the CLE can lead to interventions that may enhance CT. Therefore, the purpose of the study was to explore the optometry students’ experiences of their CLE at a National Referral and Teaching Hospital (NRTH) within an LRS. An appropriate CLE enhances the learning experience and it should be a goal of all optometric educational institutions to maximize the CL experience [10].

## METHODS

### Study design and setting

This was a qualitative study that involved undergraduate fourth year optometry students. The optometry students at MakCHS undergo CT in the outpatient department (OPD) of the eye clinic at Mulago National Referral and Teaching Hospital (MNRH) during their fourth year. The CT is eight weeks long, and all the 16 students were purposefully recruited into the study at the end of this period. MNRTH is located on Mulago Hill in the north of Kampala Capital City, west of MakCHS. It is approximately 1.8 km from the campus of MakCHS [12]. The eye clinic is staffed by Ophthalmologists, Senior Housing Officers (SHOs), Ophthalmic Clinical Officers, and Nurses, as well as faculty from MakCHS including Optometrists. The OPD offers several services including vision testing, comprehensive eye examinations, diagnosis and treatment of eye conditions, and pre- and post-operative care. The eye clinic also serves as a teaching facility for health professions students rotating in ophthalmology, from several institutions across the country.

### Data collection and analysis

Data was collected using face-to-face in-depth interviews in isolated areas at MNRTH. An experienced researcher in conducting interviews used open-ended questions followed by probing questions derived from participants’ responses to explore their experiences [13]. In the process, the principal investigator (PI), a graduate optometrist and educator, took notes of his thoughts, ideas and the non-verbal cues. The responses and reflexive notes provided the study with a deeper understanding of the participant’s experiences of the CLE in the eye clinic. Data saturation [14] was achieved after twelve interviews, and four participants were added to ensure that no information was abandoned. The interviews were audio recorded and lasted 30 to 45 minutes. Data was stored in a password protected laptop that could only be accessed by the PI.

The two researchers then transcribed the audios and cleaned the transcripts to make meaningful texts. Audios and their corresponding transcripts were named with unique identifiers for each participant. The field notes, audios and transcripts were shared with one other researcher for analysis. The three researchers separately listened to each audio against its corresponding transcript to get familiar with the data. During the process, an individual documented reflexive thoughts and interests about potential codes and/or themes in the data. In the first weekly meeting, peer debriefing was done to triangulate all their views about the data [15].

The data sets were discussed and all data was captured and tabulated into initial codes. A code manual was developed to give a detailed definition of each code. The researchers discussed the characteristics of the initial codes against the transcripts, reflexive notes and field notes to ensure consistency in the coding. Important sections of data that captured similar meanings were grouped together in a back-and-forth manner. Memos were used to record all interesting ideas and thoughts of possible themes in the data [15]. In the process, meeting minutes were captured to document all decisions made. The final table of codes and the minutes were then taken for each member to separately search for themes. Researchers were urged to be aware of their own beliefs and to document reflexive notes about their judgements and thoughts during the searching process.

In the second weekly meeting, the researchers reviewed the coded data and grouped together all codes that had similar meanings under different sub themes. Related subthemes were then categorized together to generate themes [14]. During the process, the researchers went back and forth to make sure that the themes were strongly linked to the raw data [15]. Each researcher gave his/her insights into the study findings to ensure that all aspects of the data were thoroughly analyzed. The session minutes were taken to capture all decisions. The emerging themes were then member checked to ensure that they resonated with the participants experiences [16]. In the third weekly meeting, feedback from the participants, which did not suggest any changes, was used to finalize the analysis process. Direct quotes were used to reflect participants’ experiences in their own words [16].

## RESULTS

### Participants’ demographic information

The study comprised of 16 participants with 7 females and 9 males. 13 of these were aged between 21–25 years. Participants had joined the undergraduate optometry degree program under one of two university entry schemes. Majority (n = 13) were direct entrants with a Uganda Advanced Certificate of Education while the remaining 3 held a diploma in ophthalmic clinical medicine and were admitted through the diploma entry scheme.

### Themes identified from the data.

Two themes, teaching and learning at the eye clinic and organization of the eye clinic, were identified.

### Theme A: Teaching and learning at the eye clinic

Theme A: Teaching and learning at the eye clinic were defined as educational activities and interactions that took place within the CLE of the eye clinic at MNRTH. Three sub themes, that is first day of CT, students’ roles, and learning outcomes were grouped under the theme.

### Sub theme A1: First day of clinical training.

On the first day, students received a pre-placement briefing in which faculty provided an overview of what to expect and the objectives of the CT. This was followed by an on-site tour around the eye clinic premises which was led by one of the faculty, and a hospital senior nurse. Respondents mentioned that the two sessions helped them to become familiar with the clinical setting at the eye clinic.

*We received a briefing from our lecturers. They provided us with an overview of what to expect and the objectives of the rotation* (Participant B).

*Our supervisor introduced us to individuals working at the eye clinic while the senior nurse took us through the different rooms and explained the activities and procedures carried out in each of them. This orientation gave us a better understanding of the overall setup and functions of the clinic* (Participant A).

Participants reported mixed feelings of excitement, stress, anxiety, nervousness and uncertainty on the first day. They hesitated to actively engage with hospital staff during observations in consultation rooms. Students’ emotions were attributed to a lot of expectations to perform, feelings of inferiority and the new experience at the clinic.

*On my first day at Mulago, I have to admit that I felt a bit anxious… It was a new experience for me, especially since I was just starting my clinical practice* (Participant F).

*Being surrounded by senior ophthalmologists, I couldn’t help but question my abilities as a student. Initially, I hesitated to actively engage with them, fearing that my knowledge might not be sufficient to match theirs* (Participant L).

However, they reported a gradual gain of confidence and active participation in discussions with the staff, and other activities at the eye clinic. This also improved their skills over time.

*…but as time went on, I became more accustomed to the routine and understood the limitations and expectations of the rotation* (Participant D).

*However, as time went on, I started to gain more confidence and learned how to work effectively with young patients* (Participant M).

### Sub-theme A2: Students’ roles at the eye clinic.

During their CT, optometry students engaged in patient care through hands on practice and observation from hospital staff as well as fellow students.

Respondents mentioned that they worked independently with patients under the guidance from their supervisors.

*Each of us had individual interactions with the patients, working independently to assess and manage their needs* (Participant C).

*We consult our lecturers. They are available to provide guidance and support when needed* (Participant H).

Despite individual patient interactions, the students shared their experiences by discussing different cases amongst themselves. Students described these discussions as an opportunity for learning from one another’s experiences.

*If I encounter any difficulties, I feel comfortable reaching out to them, knowing that they will be there to offer guidance or lend a helping hand* (Participant A).

*As a group, we collaborate and share our experiences, discussing different cases and learning from one another. We can collectively analyze and reflect on the cases we have seen, which enhances our understanding and knowledge in the field* (Participant E).

Students conducted a range of activities in eye care such as history taking, refractions, pupillary distance measurements, visual acuity tests, tonometry, and binocular vision assessments. They mentioned that they did not carry out slit lamp examinations, color vision and stereopsis tests while at the eye clinic of MNRTH.

*On the optometry side, we conduct procedures such as PD measurements, refractions, and binocular vision assessments* (Participant E).

*We perform preliminary tests such as visual acuity tests, pupil examinations, and tonometry. However, we don’t do slit lamp, color vision and stereopsis tests* (Participant C)

### Sub-theme A3: Learning outcomes from the eye clinic.

The CT expanded the students’ knowledge in diagnosing and managing various eye conditions. They developed skills in refraction, visual acuity testing for children, and managing binocular vision conditions; and attitudes such as empathy, effective communication, patient centered care, confidence, and decision making which are all essential for optometry practice.

*I became more proficient in performing accurate refractions. I had the chance to enhance my abilities in conducting visual acuity tests for children. […] but I learned how to handle such situations with patience and empathy* (Participant N).

*My communication skills improved as I learned how to explain the diagnosis and treatment options to patients in a clear and understandable way* (Participant F).

### Theme B: Organization of the eye clinic

Theme B: Organization of the eye clinic was defined as the systematic arrangement and management of various elements within the eye clinic to ensure efficient and effective delivery of eye care services and learning experiences. It encompasses three sub themes namely, sections of the eye clinic, management of the eye clinic and supervision.

### Sub-theme B1: Sections of the eye clinic.

The eye clinic was divided into two sections from which optometry students undertook their CT. While in the optometry section, students performed hands-on practices particularly refraction under the guidance of faculty, whereas in the ophthalmology section, they primarily observed from ophthalmologists and SHOs. Each student went through each section during the eight weeks at MNRTH.

*At Mulago National Referral Hospital, the training for optometry is divided between the optometry clinic and the ophthalmology clinic* (Participant M).

*I found the optometry clinic side more interesting because we got to do more practical tasks, such as refraction and binocular vision. On the other hand, the ophthalmology clinic provided a great opportunity to observe rare cases that we wouldn’t have seen otherwise* (Participant B).

### Sub-theme B2: Management of the eye clinic.

A consultant ophthalmologist headed the eye clinic and addressed all concerns arising. One of the lecturers of the optometry program played a crucial role in overseeing the optometry section. He was the go-to person for assistance and he ensured the smooth functioning of this section. There was a senior nurse who run the day-to-day activities of the clinic and offered additional support in both sections.

*In optometry clinic, our lecturer, Mr. ……, takes on the role of overseeing the clinic and managing its operations* (Participant I).

*Doctor …… manages the ophthalmology clinic. If we require something or have any specific concerns, we can go to him for assistance or guidance* (Participant F).

### Sub-theme B3: Supervision at the eye clinic.

Students’ supervision was provided by a mix of optometrist lecturers, hospital staff - ophthalmologists and SHOs, and optometrist interns who had recently graduated. Respondents mentioned that these were responsible for monitoring their progress and providing guidance throughout their clinical activities.

*In the optometry clinic, supervision is provided by our lecturers and interns who have finished their last semester. Both of them supervise and provide guidance during hands- on activities like refraction* (Participant K).

*Ophthalmologists’ presence and guidance ensure that we receive the necessary support and instruction while observing and participating in procedures. When they are not around, the SHOs take on this role* (Participant G).

Respondents mentioned that the supervisors were supportive, always ready to guide and correct them, and were willing to answer questions. They advised the students on approaching patient cases, managing complex situations, and shared their expertise which enhanced students’ learning experiences and helped in refining their clinical skills.

*The graduates were generally cooperative and willing to provide us with the necessary information. They would answer our questions and give us a detailed explanation* (Participant N).

*Our lecturers advised us on how to approach specific patient cases and shared their knowledge helping us navigate through challenging scenarios. Their confirmation of our measurements or observations boosted our confidence* (Participant P).

## DISCUSSION

The purpose of this study was to explore the experiences of optometry students in the clinical learning environment at the eye clinic of a national referral and teaching hospital within a low resource setting. Key findings from the study indicate that students’ clinical learning was characterized by initial feelings of anxiety and a gradual gain of confidence and skills under the support of their supervisors during patient interactions.

The findings of the current study highlight the importance of communicating the objectives of CT prior to exposing students to the CLE. Briefing sessions and other similar ways can help students in gaining the picture and expectations of the clinical setting. Such sessions give students an opportunity to ask questions and seek for clarification on areas that would otherwise be worrying to them. Anticipating the context of real-life application of theoretical knowledge in a clinical setting can cause feelings of anxiety, stress and nervousness as seen in this study. Educators should utilize their own experiences and those of previous students in CLE to effectively communicate with the aim of alleviating such feelings in students entering the CLE for the first time.

Although briefing sessions may help, educators should not assume that all these feelings can be eliminated before entry into the CLE. The findings of the current study emphasize this by demonstrating how students’ growth in a CLE is a transformative journey from initial hesitancy and feelings of inferiority to increased confidence and active engagement. With time, students become familiar with the setting and the people within the CLE which helps them to overcome personal fears and self-doubt, as well as increase interaction with individuals in the clinic. This is consistent with an Australian study by Kirkman and colleagues [9] in which students reported an initial phase of apprehension and uncertainty followed by a gradual development of a sense of comfort. Therefore, educators and supervisors within a CLE should closely monitor the students’ progress in order to identify and help students who may have difficulty in coping up with the CT.

The present study shows that learning within the clinical setting is majorly through patient interaction, and hence the outcomes of the learning process directly impact patient’s lives. Hands-on practice and observation are good approaches to CT within the CLE but it is essential that clinical educators pay attention to the order in which these methods are used. In order to achieve maximum benefit for the students’ learning while ensuring patient safety, it is appropriate that students observe from senior hospital staff and faculty before they can undertake hands-on practice. This would help students to understand the basic procedures as applied in a clinical setting as well as how to handle patients. CLEs have a common task to offer the best patient care while maintaining the maximum level of education for the students [10] yet the patients’ safety and needs are the primary focus and must be maintained [17].

In addition, a good organization and management structure is essential in ensuring an efficient and effective CLE. In a CLE, the responsibilities of students can range from clinical procedures to continuous learning and if not well managed, students may miss out on learning opportunities or patients may miss out on good care. Therefore, good organization provides a dynamic and engaging environment for learning and safety for patients. It is important that students get acquainted with the structure and functioning of the clinical setting as shown in this study so that they can appropriately address any issues occurring within the clinic. Similar findings were reported in a study by Adam et al [18] that students’ satisfaction and successful learning is likely to occur in a clinical environment that is well structured and organized.

Sending students into a CLE is likely not to achieve the intended learning outcomes of CT if students do not have proper supervision. From the current study’s findings, good supervision was a key factor in enhancing the students’ learning experiences during their CT. Supervisors should be approachable and willing to support students in different aspects of the training. When the gap between the supervisor and the student is lessened, their comments, suggestions and evaluations can significantly foster improvement in the students’ performance, help to boost their confidence, motivation, and skill-building. According to Levy, supervisors must be clinically competent, knowledgeable, and have good teaching and interpersonal skills [19]. A study by Denial at al revealed that supervisors had a key role in enhancing students’ socialization, self-confidence and professional growth [10].

Generally, the present study shows that training optometry students at an eye clinic enhances their clinical skills and development in refraction, binocular vision and communication as well as diagnosing and managing various eye conditions as observed within the clinic. Optometry educators should therefore take it upon themselves to establish partnerships with eye clinics at different levels in the health care system to expose students to a wide experience of reality as they transit into independent professional practice. The experience of students in CT is key in optometric education [9] and should be utilized fully.

## CONCLUSION

CT plays a key role in developing students’ knowledge, skills and attitudes essential for practice. The students’ experiences in a CLE take a transformative nature from initial hesitancy and feelings of inferiority, anxiety, uncertainty and nervousness to increased confidence and active engagement. It is therefore important that students’ progress is closely monitored in order to achieve the benefits of the training among all the students, which emphasizes the need for good and proper supervision. Future studies should compare optometry students’ experiences in lower-level health units to those in national referrals in order to provide the differences and similarities which would inform decisions for training students in other eye clinics within the health care system.

## Data Availability

The datasets generated and/or analysed during the current study are available in the Makerere University Institutional Repository, [http://hdl.handle.net/10570/12188].

## Abbreviations

CL: Clinical learning
CLE: Clinical learning environment
CT: Clinical training
LRS: Low resource setting
MakCHS: Makerere University College of Health Sciences
MNRTH: Mulago National Referral and Teaching Hospital
OPD: Outpatient department
SHO: Senior Housing Officer
